# Correlates of Spreading Depolarization, Spreading Depression, and Negative Ultraslow Potential in Epidural Versus Subdural Electrocorticography

**DOI:** 10.3389/fnins.2019.00373

**Published:** 2019-04-24

**Authors:** Jens P. Dreier, Sebastian Major, Coline L. Lemale, Vasilis Kola, Clemens Reiffurth, Karl Schoknecht, Nils Hecht, Jed A. Hartings, Johannes Woitzik

**Affiliations:** ^1^Center for Stroke Research Berlin, Charité – Universitätsmedizin Berlin, Corporate Member of Freie Universität Berlin, Humboldt-Universität zu Berlin, and Berlin Institute of Health, Berlin, Germany; ^2^Department of Neurology, Charité – Universitätsmedizin Berlin, Corporate Member of Freie Universität Berlin, Humboldt-Universität zu Berlin, and Berlin Institute of Health, Berlin, Germany; ^3^Department of Experimental Neurology, Charité – Universitätsmedizin Berlin, Corporate Member of Freie Universität Berlin, Humboldt-Universität zu Berlin, and Berlin Institute of Health, Berlin, Germany; ^4^Bernstein Center for Computational Neuroscience Berlin, Berlin, Germany; ^5^Einstein Center for Neurosciences Berlin, Berlin, Germany; ^6^Department of Neurosurgery, Charité – Universitätsmedizin Berlin, Corporate Member of Freie Universität Berlin, Humboldt-Universität zu Berlin, and Berlin Institute of Health, Berlin, Germany; ^7^UC Gardner Neuroscience Institute, College of Medicine, University of Cincinnati, Cincinnati, OH, United States; ^8^Department of Neurosurgery, College of Medicine, University of Cincinnati, Cincinnati, OH, United States

**Keywords:** cerebral ischemia, hypoxia, malignant hemispheric stroke, spreading depression, subarachnoid hemorrhage

## Abstract

Spreading depolarizations (SDs) are characterized by near-complete breakdown of the transmembrane ion gradients, neuronal oedema and activity loss (=depression). The SD extreme in ischemic tissue, termed ‘terminal SD,’ shows prolonged depolarization, in addition to a slow baseline variation called ‘negative ultraslow potential’ (NUP). The NUP is the largest bioelectrical signal ever recorded from the human brain and is thought to reflect the progressive recruitment of neurons into death in the wake of SD. However, it is unclear whether the NUP is a field potential or results from contaminating sensitivities of platinum electrodes. In contrast to Ag/AgCl-based electrodes in animals, platinum/iridium electrodes are the gold standard for intracranial direct current (DC) recordings in humans. Here, we investigated the full continuum including short-lasting SDs under normoxia, long-lasting SDs under systemic hypoxia, and terminal SD under severe global ischemia using platinum/iridium electrodes in rats to better understand their recording characteristics. Sensitivities for detecting SDs or NUPs were 100% for both electrode types. Nonetheless, the platinum/iridium-recorded NUP was 10 times smaller in rats than humans. The SD continuum was then further investigated by comparing subdural platinum/iridium and epidural titanium peg electrodes in patients. In seven patients with either aneurysmal subarachnoid hemorrhage or malignant hemispheric stroke, two epidural peg electrodes were placed 10 mm from a subdural strip. We found that 31/67 SDs (46%) on the subdural strip were also detected epidurally. SDs that had longer negative DC shifts and spread more widely across the subdural strip were more likely to be observed in epidural recordings. One patient displayed an SD-initiated NUP while undergoing brain death despite continued circulatory function. The NUP’s amplitude was -150 mV subdurally and -67 mV epidurally. This suggests that the human NUP is a bioelectrical field potential rather than an artifact of electrode sensitivity to other factors, since the dura separates the epidural from the subdural compartment and the epidural microenvironment was unlikely changed, given that ventilation, arterial pressure and peripheral oxygen saturation remained constant during the NUP. Our data provide further evidence for the clinical value of invasive electrocorticographic monitoring, highlighting important possibilities as well as limitations of less invasive recording techniques.

## Introduction

Spreading depolarization is the principal mechanism causing cytotoxic oedema and excitotoxicity in cerebral cortex and basal ganglia (Ochs and Van Harreveld, 1956; van Harreveld, 1959; Dreier et al., 2013, 2018a; Hinzman et al., 2015). Using ECoG, SD is observed as a large negative direct current (DC) shift (frequency band: <0.05 Hz). The SD continuum describes the spectrum from terminal SD in severely ischemic tissue to transient SDs with negative DC shifts of intermediate to short duration in tissue that is less ischemic or normal (Dreier and Reiffurth, 2015; Hartings et al., 2017b). Accordingly, SDs occur in human conditions ranging from CA, to stroke, to the harmless migraine aura (Dreier et al., 2006, 2018b; Dohmen et al., 2008; Lauritzen et al., 2011). This means that there are commonalities yet also considerable variations in SD properties along the continuum. Terminal SD, for example, is characterized by initiation of a NUP, which is similar to the negative DC shift of prolonged SD, but specifically refers to a negative potential component during progressive recruitment of neurons into cell death. The NUP has been recorded in patients dying in the wake of CA (Dreier et al., 2018b), in a patient with aSAH undergoing brain death despite continued circulatory function (Carlson et al., 2018), and in patients with ECI and DCI after aSAH (Oliveira-Ferreira et al., 2010; Dreier, 2011; Drenckhahn et al., 2012; Hartings et al., 2017c; Luckl et al., 2018).

The continuum is also manifested in the consequences of SD. In electrically active tissue, SD induces spreading depression of activity, which is measured in the AC-ECoG range (>0.5 Hz) (Dreier et al., 2017). The depression is short-lasting in normal tissue but progressively prolonged in metabolically impaired areas, such that SD can induce even persistent electrical inactivity (=isoelectricity) that develops in a spreading pattern (Oliveira-Ferreira et al., 2010). Thereafter, SDs cannot induce further spreading depression and are termed ‘isoelectric SD’ (Hartings et al., 2011). This state of electrical silence contrasts with ‘non-spreading depression,’ which is the electrical silence that develops in tissue simultaneously after the onset of severe ischemia, 1–5 min prior to terminal SD (Dreier, 2011; Dreier et al., 2018b). SD also induces tone alterations in resistance vessels, leading to either transient hyperemia (normal neurovascular response) in healthy tissue or severe hypoperfusion (inverse neurovascular response = spreading ischemia) in tissue at risk for progressive injury (Dreier et al., 1998; Shin et al., 2006). Experimentally, SI could be the sole cause of widespread cortical infarcts (Dreier et al., 2000). The continuum from SH to SI was recorded in patients with aSAH, TBI, and MHS (Dreier et al., 2009; Dreier, 2011; Woitzik et al., 2013; Hinzman et al., 2014).

Subdural recordings from platinum–iridium (Pt/Ir) electrodes have been the gold standard for intracranial monitoring of SDs in humans (Dreier et al., 2017; Eriksen et al., 2019). Despite the many translational successes achieved with these electrodes, Pt/Ir has some shortcomings as an electrode material for recording slow potentials, particularly in contrast to the Ag/AgCl-based electrodes used in animals (Masvidal-Codina et al., 2018). For instance, as a polarizable material, Pt/Ir has a capacitive nature that may result in filtering or distortion of slow potentials (Hartings et al., 2017a). Here, in an approach of reverse translation from bedside to bench (Fisher, 2013), we investigated how reliably Pt/Ir electrodes record negative DC shifts of varying duration, including the NUP, by studying the full SD continuum in rats under conditions of normoxia, hypoxia, and severe global ischemia. We then conducted a study of bench to bedside translation, based on the suggestion from animal experiments that epidural recordings are a possible semi-invasive alternative to subdural recordings (Iijima et al., 1992; Kang et al., 2013; Luckl et al., 2018). Epidural recordings are of interest as an intermediate step between invasive subdural and non-invasive scalp EEG. Although correlates of SD were clearly identified in continuous scalp EEG recordings when performed simultaneously with subdural ECoG (Drenckhahn et al., 2012; Hartings et al., 2014), scalp EEG alone may not be sufficient to reliably diagnose SDs, particularly in patients with intact skulls (Hartings et al., 2018; Hofmeijer et al., 2018). Therefore, we investigated the performance of epidural titanium peg electrodes (Barnett et al., 1990) compared to subdural electrodes in detecting SDs in patients with either aSAH or MHS. In this series, we recorded a patient who displayed an SD-initiated NUP while undergoing brain death despite continued circulatory function, as in another case reported previously (Carlson et al., 2018). The NUP was recorded not only subdurally but also epidurally, providing further insight into the nature of this phenomenon.

## Materials and Methods

### Animals

This study was carried out in accordance with the recommendations of the Landesamt für Gesundheit und Soziales Berlin (LAGeSo) (G0152/11). The protocol was approved by the LAGeSo (G0152/11). All experimental procedures were conducted in accordance with the Charité Animal Welfare Guidelines. The reporting of animal experiments complies with the ARRIVE Guidelines. Naïve, male, wild-type Wistar rats (*n* = 8; 250–400 g, ∼10–16 weeks old, supplied by Charles River, Germany) were anesthetized with 100 mg/kg body weight thiopental-sodium intraperitoneally (Trapanal, BYK Pharmaceuticals, Konstanz, Germany), tracheotomized, and artificially ventilated (Effenberger Rodent Respirator, Effenberger Med.-Techn. Gerätebau, Pfaffing/Attel, Germany). The left femoral artery and vein were cannulated. Body temperature was maintained at 38.0 ± 0.5°C using a heating pad. Systemic arterial pressure (Pressure Monitor BP-1, World Precision Instruments, Berlin, Germany) and expiratory pCO_2_ (Heyer CO_2_ Monitor EGM I, Bad Ems, Germany) were monitored. Arterial pO_2_ (p_a_O_2_), pCO_2_ (p_a_CO_2_) and pH were serially measured using a Compact 1 Blood Gas Analyzer (AVL Medizintechnik GmbH, Bad Homburg, Germany). Adequacy of the level of anesthesia was assessed with testing motor and blood pressure responses to tail pinch.

**FIGURE 1:**
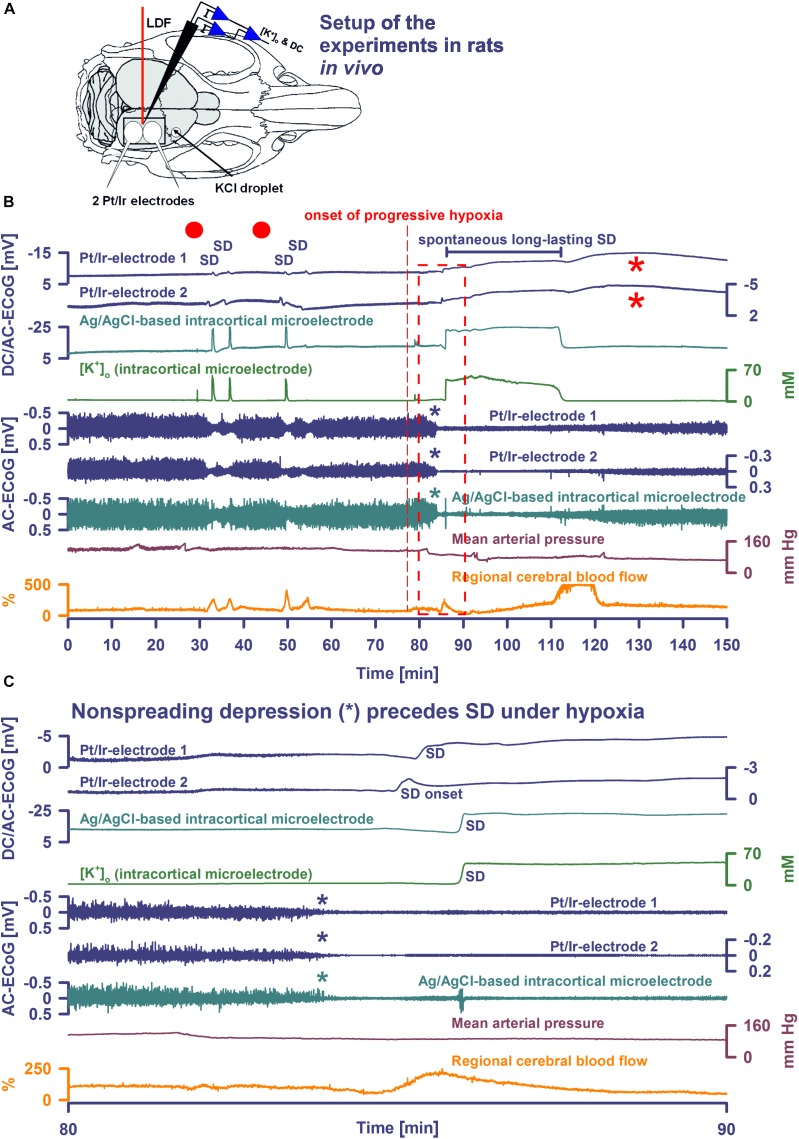
Comparison of normoxic SD with hypoxic SD preceded by non-spreading depression of spontaneous activity. **(A)** Experimental setup in rats. LDF, laser-doppler flow probe; [K^+^]_o_ and DC, K^+^-selective glass-microelectrode; Pt/Ir electrodes, platinum/iridium plate electrodes; KCl droplet, SD was triggered with a droplet containing KCl at 2M. Panel **(B)** shows the first half of an experiment with 4 KCl-induced SDs followed by the first spontaneous SD induced by hypoxia, and panel **(C)** shows the boxed portion of **(B)** with an expanded time scale. KCl application is marked with red dots.Traces 1–3: The full-band ECoG signal (DC/AC-ECoG) contains information on both the negative DC shift that identifies SD (Dreier, 2011) and the SD-induced depression of activity (negative is up for ECoG recordings shown in all figures). Note the differences in amplitude and shape of the DC shift between the gold standard recording technique in animals, the intracortical glass-microelectrode, and the Pt/Ir electrodes on the brain surface, gold standard in humans (cf. Table 1). For example, the amplitude ratio of the initial DC negativity divided by the subsequent DC positivity was significantly larger for the glass-microelectrode than for the Pt/Ir electrodes in normal tissue [Ag/AgCl: 9.1 (IQR = 8.0, 17.0); caudal Pt/Ir: 1.2 (IQR = 1.0, 1.7); rostral Pt/Ir: 1.0 (IQR = 0.7, 1.3), *P* ≤ 0.05, *n* = 7, Kruskal–Wallis One-Way Analysis of Variance on Ranks with *post hoc* Dunn test]. Also note the spread of the SD in rostral (Pt/Ir-electrode 2)-caudal (Pt/Ir-electrode 1) direction. Trace 4: The hallmark of SD is the abrupt, near-complete breakdown of the transcellular ion concentration gradients. Here, the large SD-associated increase of [K^+^]_o_ is seen. Note the significant prolongation under hypoxia. Traces 5–7: The spontaneous activity is assessed in the higher frequency band between 0.5 and 45 Hz. Note that the SD under hypoxia is preceded by non-spreading depression of the spontaneous activity [blue and green asterisks in **(B,C)**]. Non-spreading depression precludes that the subsequent SD can induce spreading depression of the activity. Interestingly, the depression of activity was reversible after the SD despite continued hypoxia. Trace 8: MAP fell under hypoxia. Trace 9: SD-induced hyperemia under normoxia and hypoxia, respectively. Note the marked reactive hyperemia following the recovery from SD under hypoxia. Note also that the Pt/Ir electrodes in contrast to the glass microelectrode displayed a marked negative DC reverberation following the recovery from SD under hypoxia (red asterisks), which may hinder correct assessment of the duration of the negative DC shift of SD. The fourth SD under normoxia showed much smaller amplitude than the three previous SDs at both the Pt/Ir-electrode 2 and the Ag/AgCl-based intracortical microelectrode. Such amplitude reductions are sometimes observed in clusters of SDs and could, for example, result from a proportion of parenchymal cells that are still refractory after the previous SD.

A parietal craniotomy was performed using a saline-cooled drill and the dura was removed. The composition of ACSF in mM was: NaCl 127.5; KCl 3.0; CaCl_2_ 1.5; MgSO_4_ 1.2; NaHCO_3_^-^ 24.5; glucose 3.7; urea 6.7 (Levasseur et al., 1975). The ACSF was equilibrated with a gas mixture containing 6.6% O_2_, 5.9% CO_2_, and 87.5% N_2_. A pO_2_ between 90 and 130 mmHg, a pCO_2_ between 35 and 45 mmHg, and a pH between 7.35 and 7.45 were accepted as physiological. rCBF was continuously monitored using LDF (Periflux 4001, Perimed AB, Järfälla, Sweden). A double-barreled K^+^-selective microelectrode was used to measure the intracortical DC/AC-ECoG and the extracellular K^+^ concentration ([K^+^]_o_) in the open window (Petzold et al., 2005; Kang et al., 2013). The cortical measuring depth was 300 μm. In addition, two Pt/Ir plate electrodes (Ad-Tech Medical, Racine, WI, United States) were placed on the cortex as shown in Figure 1A (total diameter: 4 mm, contact surface: 2 mm, distance between electrode centers: 5 mm). An Ag/AgCl electrode was placed for a reference in the neck. Electrodes were connected to differential amplifiers (Jens Meyer, Munich, Germany). Analog-to-digital conversion was performed using a Power 1401 (Cambridge Electronic Design Limited, Cambridge, United Kingdom). Systemic arterial blood pressure as well as [K^+^]_o_, DC- and AC-ECoG, rCBF and expiratory pCO_2_ were continuously recorded using a personal computer and Spike 2 software (version 6, Cambridge Electronic Design Limited).

Following surgical preparation, SDs were triggered first under normoxic conditions by applying a droplet of 2M KCl at a rostral burr hole. After cessation of SDs and recovery of baseline levels, hypoxia was then induced by gradually substituting N_2_ for O_2_ in the inspired air over ∼60 s. Thereafter, animals were monitored while hypoxia was maintained until animals died spontaneously. In some animals, additional SDs were provoked by KCl application during hypoxia.

### Statistical Analysis in General

No prior information was available which would have enabled us to perform sample size estimations based on evidence. We thus chose a sample size of eight animals which is standard in the field. For the clinical pilot study, we had originally chosen a sample size of 20 patients, but peg electrodes were no longer available (see below). Therefore, only seven patients were included. All analyses were exploratory and are reported with the caveat that no formal power consideration was performed on them. All data in text and figures is given as median and IQR. We only used non-parametric tests because of the small sample sizes. The exact statistical tests are given in the text. *P* ≤ 0.05 was accepted as significant.

### Analysis of Animal Data

Animal data was prospectively collected and analyzed by comparing relative changes of rCBF and absolute changes of DC potential and [K^+^]_o_. rCBF changes were calculated in relation to baseline (=100%) using a zero level established after death. No animals were excluded from analysis. The data was analyzed in an un-blinded fashion with LabChart-8 software (ADInstruments, Bella Vista, NSW, Australia).

### Patients

The study was carried out in accordance with the recommendations of the ethics committee of the Charité – Universitätsmedizin Berlin (EA4/175/17) with written informed consent from the patients or the patients’ legally authorized representatives. All patients or patients’ legally authorized representatives gave written informed consent in accordance with the Declaration of Helsinki. The protocol was approved by the ethics committee of the Charité – Universitätsmedizin Berlin. Results are reported in accordance with the STROBE guidelines^1^. Prospective inclusion and exclusion criteria for aSAH and MHS patients in the COSBID have been previously described (Woitzik et al., 2014; Winkler et al., 2017). Five patients with major aSAH and two patients with MHS were consecutively enrolled at Campus Benjamin Franklin, Charité – Universitätsmedizin Berlin between June 2017 and January 2018 in this pilot study.

Neurosurgeons in charge recruited patients into invasive neuromonitoring when they assumed a potential benefit for the patient based on clinical presentation and preinterventional CT scan leading them to suspect an emerging serious risk of epileptic seizures or infarct progression. Patients at high risk often require ventilation and analgo-sedation. This limits neurological assessment. Therefore, diagnosis and treatment of secondary complications are often delayed. Invasive real-time neuromonitoring can help the neurointensivist to diagnose earlier and initiate treatment earlier (Dreier et al., 2017).

In patients with aSAH, a linear electrode strip (six Pt/Ir contacts spaced at 10 mm) (Wyler, Ad-Tech Medical, Racine, WI, United States) was placed on the cortical surface of the vascular territory of the aneurysm-carrying vessel following surgery to secure the aneurysm (Dreier et al., 2006, 2009). In two patients with MHS, the electrode strip was placed on perfused, non-infarcted periinfarct tissue after decompressive hemicraniectomy (Dohmen et al., 2008; Woitzik et al., 2013). In addition, two epidural titanium peg electrodes (Ad-Tech Medical) were implanted through two small burr holes separated by a distance of 10 mm from one another and at a distance of 10 mm to the subdural strip. Peg electrodes are conically shaped with a ∼10 mm^2^ recording surface area in the epidural space. The tail of the electrodes was tunneled subcutaneously and exited 2–3 cm from the scalp incision. Technical aspects of implantation of subdural electrode strips have been described in detail elsewhere (Dreier et al., 2017).

Continuous ECoG and multimodal monitoring were performed following the COSBID recommendations while patients were in the ICU (Dreier et al., 2017). The near-DC/AC-ECoG (0.01–100 Hz) was recorded in five bipolar channels with a GT205 amplifier (ADInstruments). Data was sampled at 200 Hz and recorded and analyzed with a Powerlab 16/SP and LabChart-8 software. A subdermal Pt/Ir needle electrode on the frontal apex was used as reference. Additional monopolar, DC-coupled (0–100 Hz) recordings were performed with a BrainAmp amplifier (BrainProducts GmbH, Munich, Germany) and BrainVision Recorder software (BrainProducts GmbH). In one patient, the brain p_ti_O_2_ was measured using an intraparenchymal sensor (Licox CC1P1, Integra Lifesciences Corporation, Plainsboro, NJ, United States) (Dreier et al., 2009; Bosche et al., 2010; Hinzman et al., 2014; Winkler et al., 2017). ICP was monitored via ventricular drainage catheter (EVD) or ICP transducer (Codman or Camino Systems). The systemic arterial pressure was continuously recorded via radial artery catheter.

### Analysis of Patient Data

No patients were excluded from analysis. The data was analyzed in an un-blinded fashion with LabChart-8 software. The first 24 h period after the initial insult was denoted as ‘Day 0.’ SDs were identified by the consecutive occurrence of slow potential changes (<0.05 Hz) of neighboring ECoG channels. The negative DC shift was measured from start to end, regardless of morphology or multi-phasic appearance. In electrically active tissue, SD typically causes spreading depression of spontaneous activity in the AC-ECoG range (0.5–45 Hz) (Dreier et al., 2006). The duration of the depression period was scored beginning at the initial decrease in the integral of power and ending at the start of the recovery phase. Depression durations were scored to measure the TDDD (Dreier et al., 2017). The peak TDDD (PTDDD) was defined as the maximal value among all recording days. SDs in electrically active tissue received the epithet ‘spreading depression.’ SDs measured in a zone of electrically inactive tissue were denoted with the adjective ‘isoelectric.’

## Results

### SD in Rats Under Normal Conditions

Figure 1A shows the experimental setup. During SD in normoxic rats, Pt/Ir electrodes recorded the negative DC shift of SD, albeit with early and late positivities not observed in micropipette recordings to such an extent. SD reached the rostral Pt/Ir electrode earlier than the caudal one [delay: median: 76 (IQR: 70–83) s] corresponding to a propagation velocity of 3.9 (IQR: 3.6–4.3) mm/min. SD caused a hyperemic response to 272 (IQR: 191–286) % of baseline [duration: 118 (IQR: 110–127) s]. In more detail, the recordings using the Ag/AgCl-based intracortical glass-microelectrode and the Pt/Ir electrodes are compared in Figure 1B and Table 1.

**Table 1:** Characteristic changes along the SD continuum in rats *in vivo.*

Condition		Pre-SD DC positivity		SD DC negativity		Post-SD DC positivity		Negative DC reverberation post SD		Depression of activity	Increase of [K^+^]_o_ during SD	
							
		Amplitude (mV)	Duration (s)	Amplitude (mV)	Duration	Amplitude (mV)	Duration (s)	Amplitude (mV)	Duration (min)	Duration	Amplitude (mM)	Duration
Normoxia (*n* = 7)	Glass micro-electrode	Absent	Absent	-21.5 [(-19.4)–(-22.0)]	36 **s** (27–38)	1.8 (0.9–2.5)	92 (68–94)	Absent	Absent	x	From 3.0 (3.0–3.1) to 56.0 (46.0–61.5)	44 **s** (42–52)
	Rostral Pt/Ir electrode	Absent	Absent	-1.7 [(-1.4)–(-2.0)]	48 **s** (40–49)	1.4 (1.1–1.7)	99 (57–115)	-1.3 [(-0.8)–(-1.9)] (*n* = 4 out of 7)	11.0 (10.5–11.9) (*n* = 4 out of 7)	226 **s** (187–233)		
	Caudal Pt/Ir electrode	0.3 (0.2–0.9) (*n* = 3 out of 7)	35 (29–51)	-1.8 [(-1.4)–(-2.4)]	51 **s** (42–59)	1.9 (1.2–2.3)	89 (68–94)	-1.8 [(-1.5)–(-2.2)] (*n* = 4 out of 7)	16.7 (12.4–19.8) (*n* = 4 out of 7)	230 **s** (176–250)		
												
Hypoxia (*n* = 8)	Glass micro-electrode	1.8 (1.6–2.9) (*n* = 3 out of 8)	92 (80–136)	-17.4 [(-13.1)–(-18.3)]	**^∗^**7.9 **min** (1.4–17.7) **(*P* = 0.031)**	Absent	Absent	Absent	Absent		From 3.8 (3.6–5.0) to 51.5 (48.5–56.0)	^∗^12.8 **min** (5.9–20.7) (*P* = 0.016)
	Rostral Pt/Ir electrode	0.1 (0.1–0.2) (*n* = 4 out of 8)	28 (25–33) (*n* = 4 out of 8)	**^∗^**-3.9 [(-2.8)–(-5.9)] **(*P ≤* 0.05)**	**^∗^**8.5 **min** (2.2–17.5) **(*P* = 0.016)**	Absent	Absent	-1.9 [(-0.8)–(-7.2)] (*n* = 5 out of 8)	21.3 (12.7–22.1) (*n* = 5 out of 8)	**^∗∗^**21.0 **min** (8.5–57.2) (*P* = 0.004)		
	Caudal Pt/Ir electrode	0.3 (0.2–0.4) (*n* = 4 out of 8)	28 (23–35) (*n* = 4 out of 8)	^∗^-5.5 [(-2.9)–(-6.4)] **(*P* ≤ 0.05)**	**^∗^**9.3 **min** (2.2–18.4) **(*P* = 0.016)**	Absent	Absent	-5.0 [(-3.5)–(-5.5)] (*n* = 5 out of 8)	**^∗^**26.8 (19.1–27.4) (*n* = 5 out of 8) **(*P* = 0.032)**	**^∗∗^**21.7 **min** (10.3–54.0) (*P* = 0.004)		
												
During dying process (*n* = 8)	Glass micro-electrode	0.8 (0.6–1.0) (*n* = 4 out of 8)	47 (23–82) (*n* = 4 out of 8)	**^∗^**-14.5 [(-12.2)–(-15.5)] **(*P* ≤ 0.05)**	Terminal					Terminal	From **^∗^**7.4 (6.0–8.0) **(*P* = 0.05)** to 60.9 (57.2–68.5)	Terminal
	Rostral Pt/Ir electrode	1.2 (0.8–1.7) (*n* = 4 out of 8)	80 (44–115) (*n* = 4 out of 8)	-3.6 [(-0.2)–(-5.7)]	Terminal					Terminal	With delay gradual rise to 76.0 (73.0–78.0)	
	Caudal Pt/Ir electrode	1.2 (0.7–1.8) (*n* = 4 out of 8)	79 (47–125) (*n* = 4 out of 8)	**^∗^**-4.9 [(-3.3)–(-7.3)] **(*P* ≤ 0.05)**	Terminal					Terminal		


*Statistical comparisons were performed between SD under normoxia (control group), SD under hypoxia and the terminal SD using either Wilcoxon Signed Rank Test (two-group design) or Friedman Repeated Measures Analysis of Variance on Ranks with Dunn’s *post hoc* test (three-group design versus control) (^∗^*P* ≤ 0.05, ^∗∗^*P* ≤ 0.01). x: In recordings with microelectrodes, in contrast to Pt/Ir electrodes, the sharper negative DC shift of SD made it difficult to assess the depression periods using the integral of power. Data is given as median and IQR.*

### Hypoxia-Induced SD in Rats

Partial replacement of O_2_ with N_2_ in the inspired air caused a decrease of arterial p_a_O_2_ from 102 (IQR: 95–120) to 36 (IQR: 33–39) mmHg, of hemoglobin saturation with O_2_ from 98 (IQR: 97–98) to 51 (IQR: 43–52) %, of arterial pH from 7.37 (IQR: 7.36–7.39) to 6.99 (IQR: 6.88–7.22), of p_a_CO_2_ from 39 (IQR: 37–46) to 29 (IQR: 22–31) mmHg and of the base excess from -2.5 [IQR: (-1.4)–(-3.7)] to -23.7 [IQR: (-14.7)–(-27.3)] mM corresponding with hypoxia-induced metabolic acidosis (all *P* ≤ 0.05, *n* = 6, Wilcoxon Signed Rank Tests). MAP significantly fell from 112 to 69 (IQR: 56–98) mmHg (*P* = 0.016, *n* = 8, Wilcoxon Signed Rank Test), although high doses of intravenous noradrenalin of up to 67 (IQR: 57–77) μg/kg/min were intravenously administered to maintain the systemic circulation. Although MAP decreased under hypoxia, rCBF significantly increased from 97 to 211 (IQR: 113–248) % (*P* = 0.039, *n* = 8, Wilcoxon Signed Rank Test).

**FIGURE 2:**
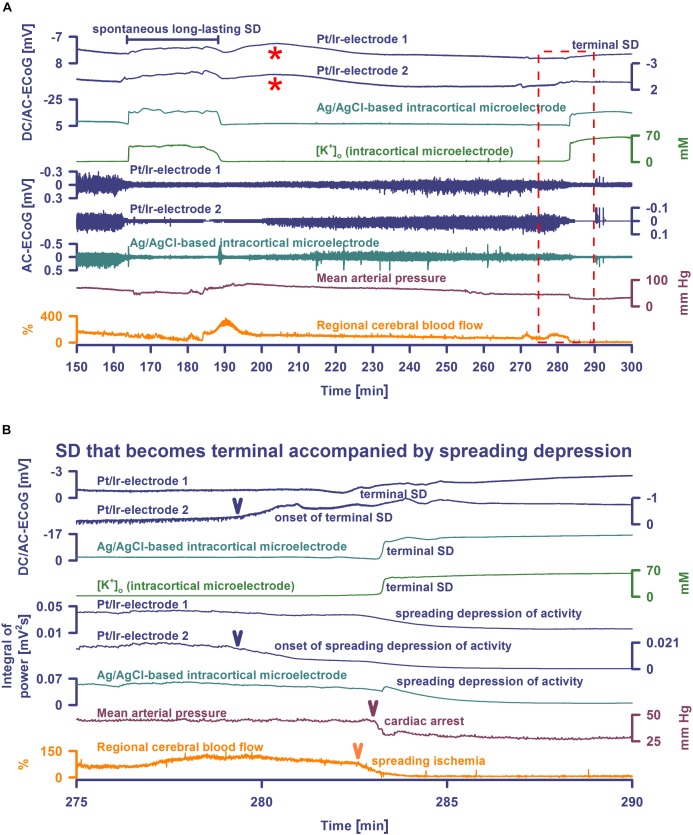
Spontaneous SD during hypoxia compared with terminal SD in death. Traces are the same as in Figure 1. The Pt/Ir plate electrodes were compared with the K^+^-selective glass microelectrode over the full SD continuum from SD in well-nourished tissue (0–80 min shown in Figure 1) to SD in hypoxic tissue to SD during global cerebral ischemia (terminal SD) (81–150 min shown in Figure 1 and 150–300 min in Figure 2). Systemic hypoxia was induced by partial replacement of O_2_ with N_2_ in the inspired air. The negative DC shift is necessary and sufficient for identification of SD, and the duration of the negativity is a measure of the metabolic and excitotoxic burden imposed on tissue by SD (Dreier et al., 2017). Accordingly, it can be seen that both negative DC shift and increase of [K^+^]_o_ were markedly longer under hypoxia than under normoxia, and they were terminal after CA. Note that the Pt/Ir electrodes in contrast to the glass microelectrode displayed a marked negative DC reverberation following the recovery from SD under hypoxia (red asterisks), which may hinder correct assessment of the duration of the negative DC shift of SD. It is also interesting in this animal that terminal SD began spontaneously under hypoxia prior to the spontaneous CA and induced spreading depression of activity. Note the spread of the terminal SD in rostral (Pt/Ir-electrode 2)-caudal (Pt/Ir-electrode 1) direction. Panel **(B)** shows the same data from **(A)** on an expanded time scale (275–290 min). The terminal SD-induced spreading depression of the activity is shown in **(B)** using the integral of power of the spontaneous activity.

**FIGURE 3:**
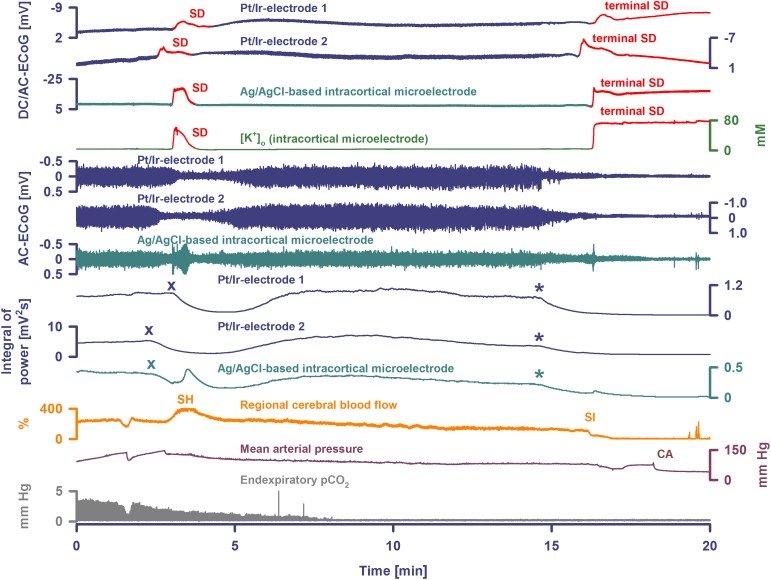
Terminal SD during death in the rat brain. Traces are similar to Figure 1, 2. A drop of KCl triggered the SD under hypoxia in this animal. Note that the hypoxic SD induced spreading depression of activity (cf. x) in contrast to the hypoxic SD in Figure 1 which was preceded by non-spreading depression. Negative DC shift and [K^+^]_o_ were only mildly prolonged indicating that hypoxia was less severe during this experiment compared to the one displayed in Figure 1, 2. Accordingly, SD induced a relatively normal spreading hyperemia (SH). Subsequently, the fraction of inspired oxygen (FiO_2_) was further reduced, and a spontaneous, slowly progressive respiratory arrest (cf. end-expiratory pCO_2_) eventually led to non-spreading depression of spontaneous activity (cf. asterisks). Thirty-five seconds after isoelectricity was established a spontaneous terminal SD occurred first at the rostral Pt/Ir electrode (rostral-caudal delay: 63 s) and triggered a marked spreading ischemia (SI) with rapid drop in rCBF from 112 to 1%. The SI was detected by the LDF probe 36 s after the SD had first appeared at the rostral Pt/Ir electrode. Circulatory arrest (CA) with a rapid drop in MAP occurred only 247 s after the onset of SI.

Spreading depolarization developed spontaneously under hypoxia in four experiments (Figure 1B,C, 2A) and was triggered by KCl application during hypoxia in the remaining ones (Figure 3). In all animals, initial SDs were transient with spontaneous recovery despite continued hypoxia. SDs induced spreading depression of activity under hypoxia in all animals except one in which non-spreading depression developed first (Figure 1C). Negative DC shifts, [K^+^]_o_ elevations, and depression periods were significantly prolonged under hypoxic compared to normoxic conditions (Table 1). Notably, Pt/Ir electrodes were able to record the SDs, but in contrast to Ag/AgCl microelectrodes, also displayed a negative DC reverberation in five of eight animals following the recovery from SD (Figure 1B, 2A and Table 1). Similar to normoxia, SD occurred with a delay of 71 (IQR: 47–100) s between the two Pt/Ir electrodes. The rCBF response to SD was impaired under hypoxia and difficult to quantify due to heterogeneity. Often, no major change was seen during SD, but a delayed hyperemic response from 211 to 368 (IQR: 215–398) % followed recovery from long-lasting SDs (Figure 1B, 2A). This was distinct from the hyperemia observed during SD in normoxic tissue. Thereafter, a relative oligemia to 135 (IQR: 76–182) % occurred (Figure 2A). The typical pattern of SI (Dreier et al., 1998; Dreier, 2011; Sugimoto et al., 2018) was not observed in any of the SDs under hypoxia that preceded the terminal one.

### Terminal SD in Rats During the Dying Process

Seven animals died spontaneously from hypoxia and one from intravenous injection of an air embolus into the femoral vein. The phase transition from life to death was characterized by four events: loss of circulation, loss of respiration, loss of spontaneous brain activity and a terminal SD without repolarization. These changes occurred in all animals, but not necessarily in the same order. Four animals had CA before terminal SD and four after. Of animals dying spontaneously under hypoxia, MAP was 45 (IQR: 43–61) mmHg at the time the first of these events occurred. When CA occurred first, brain isoelectricity developed as non-spreading depression after 71 (IQR: 49–265) s, unless the tissue was already depressed, and terminal SD began 181 (IQR: 142–302) s (*n* = 4) after CA. When terminal SD occurred first, CA followed after a median delay of 878 s (range: 23–1740, *n* = 4). In all cases, the terminal SD appeared first at the rostral Pt/Ir electrode and spread either to the caudal Pt/Ir electrode with a median delay of 60 s (range: 11–195, *n* = 7) or to the glass microelectrode (delay: 12 s, *n* = 1). This is consistent with rodent imaging experiments, which showed that the persistent SD during global cerebral ischemia would usually spread from one point of origin in a rostral-caudal direction (Farkas et al., 2008, 2010; Bogdanov et al., 2016).

**FIGURE 4:**
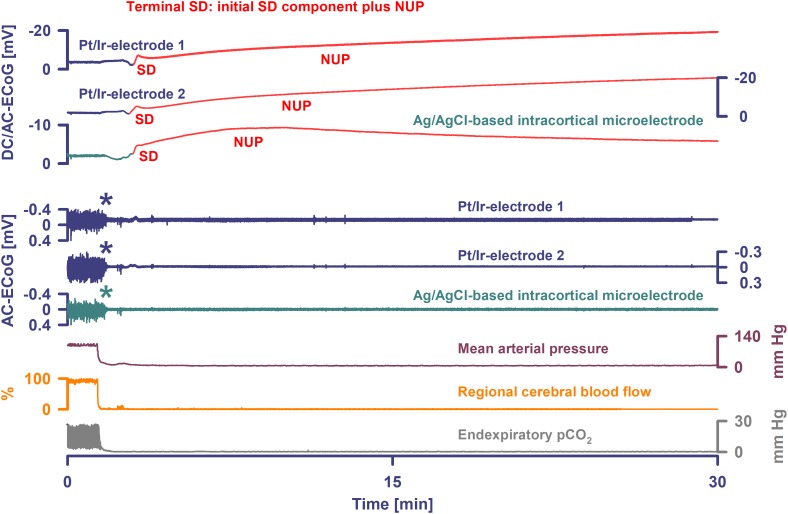
Terminal SD in the wake of circulatory arrest. Sudden circulatory arrest was induced in this rat by injection of 10 ml of air into the heart via the femoral vein. MAP fell rapidly from 99 to 6 mmHg and respiratory fluctuations of expiratory pCO_2_ ceased within 18 s; non-spreading depression caused isoelectricity within 26 s (asterisks) and terminal SD started after 98 s. Terminal SD showed an initial and a late negative DC component similar to human recordings (Dreier et al., 2018b). Whereas the initial one is the actual SD component, the late one is termed the NUP.

In four animals, isoelectricity developed in a non-spreading pattern and was followed by terminal SD. For instance, following the air embolus in one animal, MAP fell rapidly from 99 to 6 mmHg and respiratory fluctuations of expiratory pCO_2_ ceased within 18 s; non-spreading depression caused isoelectricity within 26 s and terminal SD started after 98 s (Figure 4). In two other animals, progressive CA spontaneously developed with fluctuating drops in MAP and rCBF, and was followed by non-spreading depression and then terminal SD. In yet another case (Figure 3), non-spreading depression developed minutes after respiratory insufficiency and was then followed by terminal SD, which induced SI. Thereafter, CA developed.

The remaining animals showed diverse patterns in the development of isoelectric brain activity during the dying process. In one case, the terminal SD started when MAP was 44 mmHg, the respiratory rate was 71/min, and spontaneous brain activity was still present. The SD thus triggered a spreading depression that persisted through death (Figure 2B). 179 s after SD first appeared at the rostral Pt/Ir electrode, the LDF probe detected a marked SI with sharp drop of rCBF from 81 to 0%. Systemic CA and respiratory arrest started 23 and 42 s, respectively, after the onset of SI. In two other animals, terminal SDs started when the spontaneous activity was already depressed after previous SD-induced spreading depressions. These did not cause any obvious change in rCBF, which remained at 70 and 95%, respectively. Systemic circulatory and respiratory arrest, with simultaneous drop in rCBF, did not occur until 29 min later in one case, and 27 min in the other. The electrophysiological pattern was the same in the last animal, but terminal SD was preceded by progressive circulatory failure.

### Epidural Recordings of SDs in Patients

Demographic details are given in Table 2. The total near-DC/AC-ECoG recording time from the cortical surface was 1117.5 h in the five patients with aSAH and 178.1 h in the two patients with MHS. Three patients had no SDs, but assessment was limited in one of these because recordings were artifact-laden. Three patients with aSAH had 98 SDs and one patient with MHS had 5 SDs. For further analysis, 808.0 h of simultaneous subdural DC/AC-ECoG and epidural DC/AC-ECoG recording time of sufficient quality was available during which 74 of the 103 SDs occurred. Ten of these occurred in patient 1, 16 in patient 4, 3 in patient 6, and 45 in patient 7. Seven of the 45 SDs in patient 7 occurred as part of the terminal cluster and are described further below.

**Table 2:** Summary of demographic, treatment, and SD-related data of the patients.

aSAH (subdural electrode strip, bipolar near-DC/AC-ECoG recordings)												
**No.**	**Age range**	**Lesions**	**WFNS grade**	**Modified Fisher grade**	**Location of aneurysm**	**Intervention**	**Location of electrode**	**Total number of SDs**	**PTDDD (min)**	**Peak number of SDs/24 h**	**Peak number of iSDs/24 h**	**Recording time (h)**

1	80–85	Lesions remote from strip; early: ICH (P, l), PCA infarct (r); delayed infarcts: AchA (l), MCA (l), PCA (l); delayed lacunes: ACA (r), MCA (r), PICA (b), SCA (b)	5	4	MCA	Aneurysm clipping, EVD left frontal	F (l)	10	96.8	8.3	0.0	270.5
3	70–75	Lesions remote from strip; early: ICH (F, T, r)	5	3	MCA	Aneurysm clipping, EVD right frontal	F (r)	0	0.0	0.0	0.0	188.8
4	60–65	Delayed lesion close to strip; early: PCA infarct (l), lacunes: ACA (r), MCA (l), PCA (r); delayed infarcts: ACA (b)	5	4	ACoA	Aneurysm clipping, EVD left frontal	F (r)	16	259.8	6.3	3.0	191.1
5	60–65	Early lesion close to strip; early: ACA/MCA infarcts (l), ICH (T, l), SDH hem (l)	5	3	MCA	Aneurysm clipping	P (l)	Artifact-laden				278.4
7	40–45	Delayed lesion close to strip (Figure 8); early: ACA infarct (l), ICH (P, l); delayed infarct: MCA (l)	4	4	PCoA	Aneurysm clipping, EVD right frontal	F (l)	72	1440.0	23.9	12.5	188.6

**MHS (subdural electrode strip, bipolar near-DC/AC-ECoG recordings)**												

**No.**	**Age range**	**Lesions**	**Infarct etiology**		**Intervention**		**Location of electrode**	**Total number of SDs**	**PTDDD (min)**	**Peak number of SDs/24 h**	**Peak number of iSDs/24 h**	**Recording time (h)**

2	46–50	Territorial infarct MCA (r)	Suspicion of cardiac embolism due to NSTEMI		Decompressive hemicraniectomy		P, O (r)	0	0.0	0.0	0.0	78.6
6	70–75	Territorial infarct MCA/PCA (r)	Cardiac embolism due to atrial fibrillation		Decompressive hemicraniectomy		F (r)	3	37.9	3.4	0.0	99.5


*iSD, isoelectric SD; PTDDD, peak total SD-induced depression duration of a recording day; WFNS, World Federation of Neurosurgical Societies; f, female; m, male; l, left; r, right; b, bilateral; hem, hemisphere; F, frontal; O, occipital; P, parietal; T, temporal; EVD, extraventricular drainage; ICH, intracerebral hemorrhage; NSTEMI, non-ST elevation myocardial infarction; SDH, subdural hematoma; ACA, anterior cerebral artery; AChA, anterior choroidal artery; ACoA, anterior communicating artery; PCoA, posterior communicating artery; MCA, middle cerebral artery; PCA, posterior cerebral artery; PICA, posterior inferior cerebellar artery; SCA, superior cerebellar artery.*

The negative DC shift’s peak-to-peak amplitude and duration of the remaining 67 SDs were 5.7 (IQR: 3.9–7.3) mV and 4.2 (IQR: 3.6–5.8) min. The AC-ECoG activity declined from 100 to 19 (IQR: 15–28) % during the SD-induced spreading depression. Median depression duration was 13.2 (IQR: 7.5–21.8) min. Median interval between SDs was 91 (IQR: 49–316) min.

**FIGURE 5:**
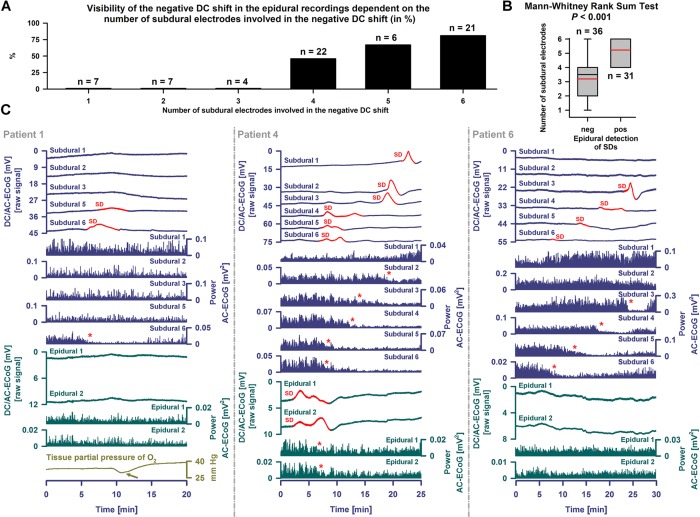
Epidural and subdural recordings of single SDs. **(A)** The 31 epidurally identified SDs involved more subdural electrodes than the 36 SDs that could not be identified using epidural recordings. **(B)** When the SDs were pooled this was statistically significant (Mann–Whitney Rank Sum Test, *P* ≤ 0.001). The whiskers (error bars) above and below the box indicate the 90th and 10th percentiles. The red reference line marks the mean. **(C)** SDs are observed as a negative DC shift propagating between neighboring electrodes (cf. top traces showing the DC/AC-ECoG (0–45 Hz) at the different electrodes, negative up). Red portions indicate potentials associated with SDs. SD-induced spreading depression is observed in the AC-ECoG recordings (0.5–45 Hz) as a rapidly developing reduction in the amplitudes of spontaneous activity that spreads together with SD between adjacent recording sites. The squared spontaneous activity is called AC-ECoG power (cf. Power AC-ECoG-traces from the different electrodes). In patient 1, 10 non-clustered SDs were recorded using subdural electrodes. Six SDs only occurred at electrode 6 and 4 also involved electrode 5. The left panel shows an example of one of these SDs. The red asterisk marks the SD-induced depression of activity at electrode 6. The light green arrow points at the SD-induced tissue hypoxia (Dreier et al., 2009; Bosche et al., 2010; Winkler et al., 2017). Different from the subdural recordings, the epidural ones did not allow clear identification of the SD, though there could be a correlate. By contrast, in patient 4 (middle panel), 14 of 16 SDs were not only seen subdurally but also epidurally. A non-clustered SD is displayed. The preceding SD occurred 12.5 h before and the subsequent one 25.1 h after this SD. Note that the SD involved all six subdural electrodes. At the epidural electrodes, not only the negative DC shift but also the SD-induced depression was visible. The red asterisks mark the SD-induced spreading depression of activity at subdural electrodes 2–5 and the two epidural electrodes. In patient 6 (right panel), the subdural DC/AC-ECoG of 3 SDs was available to the analysis. None of these showed an unambiguous correlate in the epidural DC/AC-ECoG. One of these is displayed here as an example.

**FIGURE 6:**
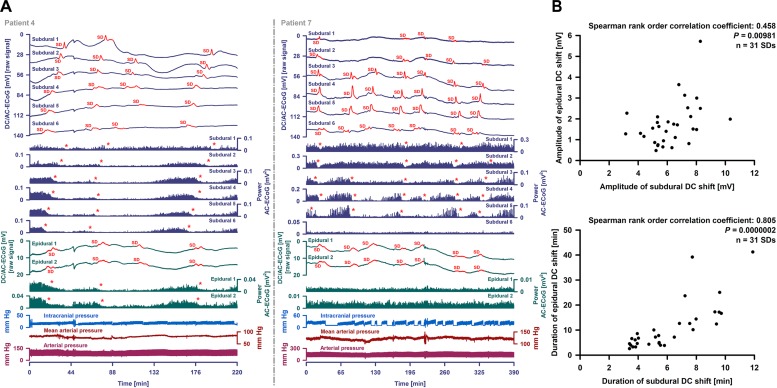
Epidural and subdural recordings of clustered SDs. **(A)** Top six traces show the raw DC/AC-ECoG recordings from each subdural electrode (negative up). Red portions indicate potentials associated with SDs. SD-induced spreading depressions are assessed in the power of the AC-ECoG (traces 7–12). Below, the epidural recordings are shown. In the left panel, clustered SDs in patient 4 are displayed. Between an MRI on Day 2 and a CT on Day 9, the patient developed a new infarct in the vicinity of the recording strip. The SDs involved all subdural and epidural electrodes. The red asterisks mark the SD-induced spreading depressions. The third SD is an isoelectric one. In patient 7 (right panel), clustered SDs on Day 8 are shown. The electrodes were located close to a delayed cerebral infarct as shown in Figure 8. The SDs were observed both subdurally and epidurally in contrast to the SD-induced spreading depressions which were only seen at the subdural electrodes. **(B)** Amplitudes and durations significantly correlated between subdural and epidural negative DC shifts indicating SDs.

With visual inspection of epidural DC-ECoG recordings alongside subdural recordings, negative DC shifts were identified for 31/67 (46%) SDs (Figure 5C). All 31 epidurally identified SDs occurred in patient 4 [14/16 SDs (88%)] and patient 7 [17/38 SDs (45%)] who had clustered SDs following COSBID’s current working definition of a cluster that at least three SDs occurred within three or fewer consecutive recording hours (Dreier et al., 2017) (Figure 6A). When the 67 SDs were pooled, the 31 epidurally identified SDs involved significantly more subdural electrodes than the 36 SDs that could not be identified in epidural recordings (Mann–Whitney Rank Sum Test, *P* ≤ 0.001, Figure 5A,B). Intervals between subsequent SDs were not significantly different between these two groups, but the subdural negative DC shift of epidurally identified SDs was significantly larger [5.4 (IQR: 3.9–7.8) mV versus 4.0 (IQR: 3.3–4.6) mV, Mann–Whitney Rank Sum Test, *P* ≤ 0.001].

Subdural negative DC shifts were larger and shorter than epidural ones [6.1 (IQR: 5.3–7.5) versus 1.5 (IQR: 1.0–2.1) mV and 5.4 (IQR: 3.9–7.8) versus 7.2 (IQR: 4.6–13.5) min, *n* = 31, Wilcoxon Signed Rank Tests with *P* ≤ 0.001]. Amplitudes and durations significantly correlated between subdural and epidural negative DC shifts (Figure 6B). Sometimes, a delay was noted between the onsets of the negative DC shift at the two epidural electrodes but this was difficult to quantify. SD-induced spreading depression of epidural AC-ECoG activity was only identified during 19 SDs (28%). In these, the decline in epidural AC-ECoG activity was slightly less pronounced than the subdural one [20 (IQR: 15–38) % versus 18 (IQR: 13–22) %]. Both activity decline (Spearman Rank Order Correlation Coefficient: 0.599, *P* = 0.00683) and duration of spreading depression (Spearman Rank Order Correlation Coefficient: 0.898, *P* = 0.0000002) significantly correlated between epi- and subdural recordings.

### A Case of Brain Death Recorded With Subdural and Epidural Electrodes

Patient 7 (cf. Table 2) was a 45-year-old woman with aSAH who presented with a WFNS score 4 on admission. The initial CT on Day 0 showed a grade 4 on the modified Fisher scale. CT angiography (CTA) revealed an aneurysm of the posterior communicating artery which was successfully treated with clip ligation on Day 0. After surgery, she was transferred to the ICU where prophylaxis of DCI with oral nimodipine commenced. The postoperative MRI scan on Day 1 showed widespread subarachnoid clots with oedema of the adjacent cortex. Laminar infarcts were seen at the frontal base, along the interhemispheric fissure and around a clot in the Sylvian fissure on the left side. No important parenchymal changes were noted on the CT scan on Day 3, but the CTA demonstrated signs of proximal vasospasm. Therefore, endovascular spasmolysis was performed. CT on Day 5 revealed progression of the cortical oedema. On Day 7, a wide and fixed left-sided pupil led to an emergency CT. This revealed a new infarct in the left MCA territory and a midline shift of 8 mm. Midazolam was added to the sedative regimen of propofol and sufentanil because of the increased ICP. On Day 9, another CT revealed progression of the infarct, 12 mm midline shift and beginning subfalcine herniation.

**FIGURE 7:**
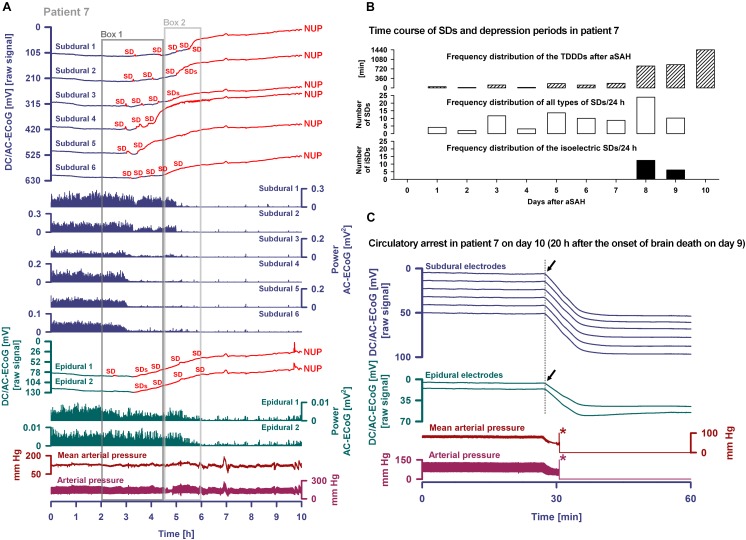
Progress to brain death in patient 7. **(A)** The six top traces show a series of several transient SDs followed by terminal SD. The terminal SD consists of the initial SD component followed by the NUP (Dreier et al., 2018b). Traces 7–12 display the SD-induced spreading depression that persisted through death (observed as a reduction in the amplitudes of the AC-ECoG power propagating from electrode to electrode). SDs and NUP are smaller in the epidural than the subdural DC/AC-ECoG recordings. However, in particular the NUP is clearly visible also in the epidural recordings (traces 13 and 14). Also the terminal SD-induced depression of activity is seen at the epidural recordings (traces 15 and 16). Traces 17 and 18 display MAP and arterial pressure fluctuations. Regions of Box 1 outlined by the dark gray lines and of Box 2 outlined by the light gray lines are shown in detail in Figure 8 which additionally gives the continuous ICP recordings. **(B)** The occurrence of SDs, SD-induced depression periods and isoelectric SDs in patient 7 over the whole clinical course. **(C)** When the patient underwent circulatory arrest 20 h after brain death the DC potential at all subdural and epidural electrodes showed a slow positive drift (positive down). The asterisks mark the moment when the arterial pressure monitoring was switched off.

**FIGURE 8:**
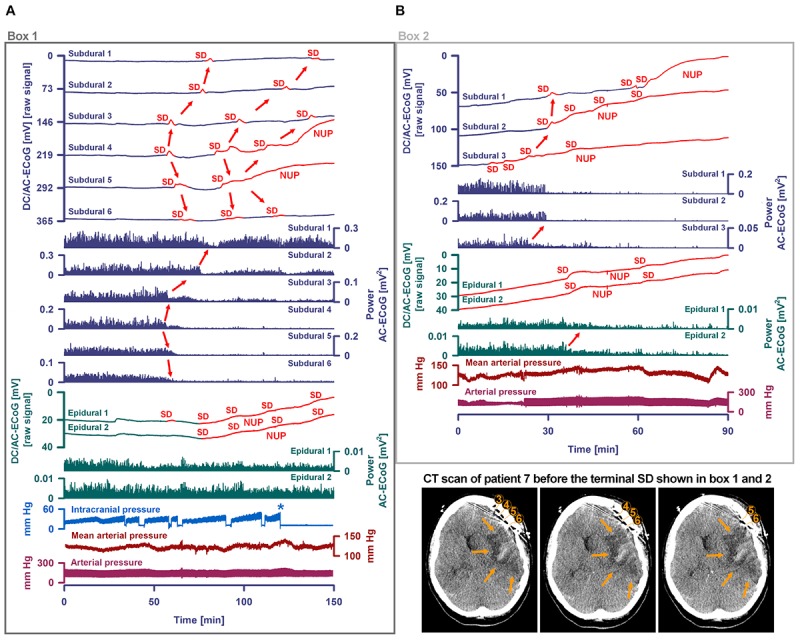
Progress to brain death in patient 7. **(A)** A subset of the same traces in Figure 7 is shown in greater detail. In addition, ICP is given. As outlined by Box 1 in Figure 7, the initial time period around the terminal SD is displayed. The SDs are marked in red. The second SD is a terminal one that starts at electrode 4 from where it spreads to electrode 5. The terminal nature of this event is indicated by the persistence of the negative DC shift. This negative potential develops further through time as a NUP, which is thought to reflect the progressive development of tissue necrosis. The red arrows mark the spreading depression of activity (middle traces). Whereas the spontaneous activity transiently recovered at subdural electrodes 1–3, the activity depression persisted through death at subdural electrodes 4–6 after the first SD. The continuous physiological variables in the bottom traces remained stable during the terminal SD. The asterisk marks the moment when the EVD was left open to counteract the ICP increase. **(B)** As outlined by Box 2 in Figure 7, the time period following the one in **(A)** is shown. The terminal SD spread further to subdural electrode 3 to 2 to 1. The activity depression at the subdural electrodes was eventually followed by activity depression at the epidural electrodes which persisted through death. Note that the NUP was superimposed by additional SDs of decreasing amplitude. In the right lower corner, three neighboring slices of the last CT scan are given, showing the subdural electrodes anterior to the delayed infarct in the MCA territory.

Occurrence of SDs, spreading depressions and isoelectric SDs during the clinical course are illustrated in Figure 7B. Clustered SDs on Day 8 after the initial hemorrhage are shown in the right panel of Figure 6A. Notably, when the last CT scan was performed on Day 9, the subdural electrode strip was still on viable cortex anterior to the infarct in the left MCA territory (Figure 8). Eleven hours thereafter, the last cluster of SDs started and led to a spreading depression with persistent loss of cortical activity through death as shown in Figure 7A. At all electrodes, SD then became terminal, since the negative potential shift, termed NUP, was persistent (20+ h). Additional SDs with reduced amplitude occurred superimposed on the NUP, similar to previous cases of focal or global brain death with maintained systemic circulation (Oliveira-Ferreira et al., 2010; Carlson et al., 2018; Luckl et al., 2018), but in contrast to cases when brain death followed systemic CA (Dreier et al., 2018b) (Figure 8). The NUP had an amplitude of -150 [IQR: (-136)–(-157)] mV at the subdural electrodes, and was also recorded at the two epidural electrodes (median amplitude: -67 mV).

At onset of the terminal cluster, MAP was at 120 mmHg, the heart rate was around 65 beats/min and the peripheral O_2_ saturation >98%. ICP was around 30 mmHg with a trend toward slow increase. The arterial blood gas analysis was normal, including serum lactate at 6 mg/dl, serum glucose at 155 mg/dl and normal serum electrolytes. However, shortly after onset of the terminal cluster, the patient was found to have an abrupt loss of brainstem reflexes with fixed dilated pupils. Serum lactate then increased to 12 mg/dl and serum glucose to 211 mg/dl; a sinus tachycardia up to 125/min developed. Following discussion with the family regarding the poor prognosis, the patient was terminally extubated 20 h later and expired shortly thereafter. Similar to the previous case of Carlson et al. (2018), the exact cause of death remained unclear given that there was no massive ICP increase suggestive of large stroke or oedema, and cardiac, pulmonary, and renal function were thought to be adequately supported. During CA at 20 h after brain death, a slow positive DC drift occurred which stabilized at 53 (IQR: 52–54) mV subdurally and 43 (IQR: 42–44) mV epidurally. No SDs were superimposed on this potential, consistent with the prior diagnosis of brain death and in contrast to cases where CA is the cause of subsequent brain death (Dreier et al., 2018b).

## Discussion

### Terminal SD Under Progressive Hypoxia in Rats

The animal experiments confirmed the characteristic changes along the full SD continuum. Specifically, the duration of depolarization and near-complete breakdown of ion homeostasis, as indicated by the negative DC shift and increase of [K^+^]_o_, varied from short-lasting under normoxia to long-lasting under systemic hypoxia to persistent under severe global ischemia, since repolarization requires activation of energy-dependent membrane pumps such as Na, K-ATPases (LaManna and Rosenthal, 1975; Major et al., 2017). Short-lasting DC shifts thus indicate sufficient ATP at the recording site to fuel fast repolarization. This feature renders the negative DC shift duration a useful measure for (a) the tissue energy status and (b) the risk of injury (cytotoxic oedema and excitotoxicity) at the recording site as reviewed recently (Dreier et al., 2017; Hartings et al., 2017b).

A significant proportion of SD waves observed in patients have intermediate characteristics, as opposed to the two extremes of the SD wave in either severely ischemic or normal tissue (Dreier and Reiffurth, 2015; Dreier et al., 2017; Hartings et al., 2017b). In patients, SD waves of intermediate character might typically indicate moderate focal ischemia at the recording site. This interpretation is consistent with previous experimental observations in brain slices (Somjen, 2001) and also with the intermediate SDs recorded here in rats under systemic hypoxia. The uniformity of intermediate SDs, no matter whether they are recorded during focal ischemia or under systemic hypoxia, indicates that energy depletion is the decisive factor producing this pattern rather than, for example, diffusion of chemicals from the core of focal ischemia to the penumbra.

In contrast to the intermediate type, persistent SDs are those that are indefinitely prolonged at the recording location and will not repolarize unless oxygen and glucose availability improve. These typically occur following non-spreading depression of activity after onset of anoxia/severe ischemia, and thus historically are termed ‘anoxic SD’ (van Harreveld, 1946; Leão, 1947; Bures and Buresova, 1957; Marshall, 1959). However, SD can transform into intermediate and persistent types even in well-nourished tissue by the mechanism of SI, which reflects inverse neurovascular coupling (Dreier et al., 1998, 2001; Dreier, 2011; Sugimoto et al., 2018). Here, we observed SI in response to terminal SD in two out of four hypoxic animals in which the terminal SD preceded the CA. This corresponds well to previous observations under hypoxia (Sonn and Mayevsky, 2000; Sukhotinsky et al., 2008). Nonetheless, terminal SD did not cause any change in rCBF in the remaining two animals, and rCBF declined only when the systemic circulation ceased. These results evidence the continuum of hemodynamic responses to SD, which ranges from the normal hyperemic response to an increasingly inverse ischemic one (Dreier et al., 2009, 2018a).

Patterns in the development of electrocerebral silence during the dying process varied in the animals. The terminal SD could be preceded by non-spreading depression of activity, or it could trigger a spreading depression that persisted through death, or it could start when the spontaneous activity was already depressed after previous SD-induced spreading depressions. In rats, the pathophysiology of hypoxic-ischemic brain injury thus shows significant variations. In part, this may reflect whether the underlying condition is primarily hypoxic/anoxic versus abrupt circulatory collapse versus prolonged hypoperfusion. Nonetheless, the phase transition from life to death is always characterized by four events: loss of circulation, loss of respiration, loss of spontaneous brain activity and terminal SD. The latencies and even the order of the four events can vary dependent on the specific etiologies and circumstances of death.

The patterns observed in the present case and in 10 cases previously reported (Carlson et al., 2018; Dreier et al., 2018b) suggest that human death is characterized by similar variations. This is important not only for research on treatment strategies of cardiac arrest and stroke that may complement efforts to re-establish circulation, but also for the debate on organ donation after (a) either cardiocirculatory death (DCD), where death is declared between a few seconds and 10 min following the cessation of circulatory function (Boucek et al., 2008; Dhanani et al., 2012; Stiegler et al., 2012; van Veen et al., 2018), or (b) brain death.

With regard to the terminal SD, it should be noted that propagation is not really the decisive property of SD and that depolarization of neurons can also take place in many other contexts. The main feature of SD is the abrupt, almost complete collapse of the small molecule gradients across cell membranes, which in the case of terminal SD is persistent and eventually leads to cell death under this condition.

### Detection of SDs Using Subdural Electrodes

The animal experiments confirmed that subdural Pt/Ir electrodes reliably identify the full SD continuum. However, reliable measurement of negative DC shift durations may be complicated by multiple sensitivities of Pt/Ir electrodes, particularly in hypoxic conditions. Here, for instance we observed negative DC reverberations on Pt/Ir electrodes that were not present in Ag/AgCl electrodes. This pattern can also occur in human recordings (Drenckhahn et al., 2012). The underlying process is unclear, but may be related to the strong catalytic capacity of platinum in contrast to Ag/AgCl for many chemical reactions. To give but two examples: (a) platinum’s catalytic effect on the redox reaction between hydrogen ions and molecular hydrogen renders the platinum-based hydrogen electrode method a standard among the various methods for measuring pH; (b) an additional reaction catalyzed by platinum is the formation of water from molecular O_2_ and hydrogen ions (Meunier and Popovic, 1988).

### Detection of SDs Using Epidural Electrodes

A number of previous animal studies suggested that SD’s negative DC shift can also be recorded using epidural electrodes (Iijima et al., 1992; Kang et al., 2013). Though subdural and intraparenchymal Pt/Ir electrodes show a good safety profile in humans (Stuart et al., 2010; Drenckhahn et al., 2016), at least theoretically, epidural electrodes pose a lower risk of bleeding and infection because they do not penetrate dura or brain (Beleza et al., 2010). However, here, only 46% of the subdurally recorded SDs could be identified using epidural electrodes. This relatively small yield may have resulted from the fact that only two epidural peg electrodes were used in each patient, in contrast to six electrodes on the subdural strip. This is supported by a previous analysis showing that a single recording location failed to detect 43% of all SDs that were identified based on consideration of a full six-electrode subdural strip (Hartings et al., 2017a). In addition, the ability to detect SDs using epidural electrodes depended on the features of a given SD. Thus, epidural electrodes were more likely to detect SDs when subdural negative DC shifts were longer and more subdural electrodes were involved in a given SD. Nonetheless, it is noteworthy that the DC shifts of SD were captured in titanium electrode recordings, since little is known about these electrodes in terms of interferences and DC recording properties. This deserves further study, not the least because titanium could also be used for intracranial recordings and is much less costly than Pt/Ir.

In a recent study, scalp EEG recordings in the rat failed to detect the negative DC shifts of SDs (Luckl et al., 2018). This supported the interpretation that DC-EEG shifts are only observable in human scalp recordings when there is an existing craniotomy (Drenckhahn et al., 2012; Hartings et al., 2014). For comparison, the median scalp DC-EEG amplitude was one twentieth of the subdural DC-ECoG amplitude (Drenckhahn et al., 2012), whereas the epidural amplitude in the present study was one quarter. The superiority of epidural peg electrodes over scalp EEG electrodes and their inferiority to subdural electrodes is congruent with findings from electrographic seizure detection (Beleza et al., 2010; Benovitski et al., 2017).

In epidural recordings, the SD-induced depression periods were even more difficult to detect than the negative DC shifts. The lower sensitivity of epidural than subdural electrodes to detect depression periods may be due to superposition of volume-conducted signals from more widespread cortical generators, due in particular to the spatial filtering of the dura. That is, epidural electrodes record from widespread sources including unaffected cortical regions that may mask and overwhelm smaller regions affected by spreading depression, making detection more difficult.

### Limitations

The typical indication for the use of epidural peg electrodes in patients is the detection of epileptic foci. Unfortunately, the demand for epidural peg electrodes is low. For this reason, the manufacturer has recently discontinued their production. Therefore, we could not include more patients and the small size of the study is a limitation. However, our results provide guidance for planning future studies. For example, the next step could be to investigate whether depth electrodes placed epidurally instead of in the parenchyma are sufficient to detect SDs.

### Terminal SDs Cause Electrocortical Silencing Prior to Clinical Brain Death

Similar to the previously reported case of Carlson et al. (2018), patient 7 progressed to brain death despite continued circulatory function. The neuromonitoring confirmed that (a) a series of SDs preceded the clinical signs of brain death, (b) SD-induced spreading depression initiated the final electrical silence recorded over the following 20 h, and (c) the terminal electrographic event was an SD, which initiated a NUP. The NUP is the largest bioelectrical signal ever recorded from the human brain and in the present case, similar to previous ones, had an amplitude of -150 mV (Carlson et al., 2018; Luckl et al., 2018). It is possible that the Pt/Ir-recorded NUP is not purely a biopotential, but results also in part from platinum sensitivities to diffusible chemicals. Nonetheless, the fact that the NUP was measured not only by the subdural but also the epidural electrodes affirms that the human NUP is a bioelectrical field potential. The epidural environment was likely unchanged with respect to signal-contaminating factors during the NUP, since systemic variables such as ventilation, MAP, and peripheral oxygen saturation stayed constant and the dura provides insulation from changes in the subdural compartment.

A clinical implication of this finding is that epidural electrodes may be sufficient to record the NUP. This could have relevance to patients who are at risk for secondary ischemia, but for whom placement of a subdural strip is not possible, due to lack of clinical indication for a craniotomy or a burr hole. Notably, the NUP occurs not only in brain death but also during ECI and DCI in aSAH patients (Oliveira-Ferreira et al., 2010; Dreier, 2011; Drenckhahn et al., 2012; Hartings et al., 2017c; Luckl et al., 2018). ECI and DCI predominantly affect the cerebral cortex (Birse and Tom, 1960; Stoltenburg-Didinger and Schwarz, 1987; Neil-Dwyer et al., 1994; Dreier et al., 2002; Rabinstein et al., 2005; Hartings et al., 2017c). A significant proportion of these ischemic events could thus be detectable by epidural recordings. A limitation might be that ECI- and DCI-associated NUPs are localized phenomena (Luckl et al., 2018) in contrast to the NUP during brain death (Carlson et al., 2018) or CA (Dreier et al., 2018b). Thus, recordings at multiple sites would be necessary because the exact location of future developing pathology is unknown when the neurosurgeon implants neuromonitoring devices.

When the patient underwent CA 20 h after brain death, a slow positive DC drift occurred. This was previously observed in cases of CA leading to brain death, where it occurred simultaneously with the parenchymal p_ti_O_2_ decline as measured by an O_2_ sensor (Dreier et al., 2018b). The present findings suggest that this drift does not reflect a cerebral field potential of biological origin but rather results from interferences, since neither brain parenchyma nor BBB should be able to generate field potentials 20 h after brain death. Consistent with this conclusion, no terminal SD was superimposed on the slow positive DC drift, in contrast to previous cases in which the brain was viable at the time of CA (Dreier et al., 2018b). The lack of terminal SD at this time, in addition to the clinical signs such as loss of brainstem reflexes and wide dilated pupils, further supports the conclusion that brain death occurred with the final cluster of SDs and associated NUP, 20 h earlier.

One remaining question is why the NUP can be at least 10 times larger in humans than in rats. The answer may relate to the different recording conditions of a closed subdural compartment in patients versus an open compartment in rats, since current leaks from the intended circuit may have occurred in the latter condition. Another possibility is that the NUP partially reflects the DC potential across the BBB and is caused by the profound interstitial and rCBF changes during the dying process. In this hypothesis, the zonal DC shift of SD, resulting from neurons and to a lesser extent from astrocytes (Makarova et al., 2008; Dreier et al., 2013), could be superimposed on a DC drift across the BBB. This hypothesis is indirectly supported by previous reports on profound differences in electrogenesis across the BBB between rats, rabbits, goats and dogs on the one hand and cats and non-human primates on the other (Tschirgi and Taylor, 1958; Held et al., 1964; Besson et al., 1970; Woody et al., 1970; Messeter and Siesjo, 1971; Kang et al., 2013). This notion may imply that future studies to uncover the generators of the NUP in humans should be performed in higher mammals rather than rodents. For example, swine share many features with humans in the pathogenesis of ischemic lesions after aSAH and would be interesting candidates for further study (Hartings et al., 2017c).

However, when considering various possible DC potential components that could contribute to the human NUP, it should not be forgotten that the NUP is experimentally defined not by simple quantifiable parameters such as its amplitude, but by three other more complex properties: (i) the beginning with SD, (ii) the fact that the ion shifts and cell oedema do not fully recover during this extremely long DC negativity, and (iii) the death of neurons (Dreier, 2011; Luckl et al., 2018). In this operational definition, however, it should be noted that the distinction of a NUP from a long-lasting SD, which has not exceeded the commitment point (Dreier and Reiffurth, 2015) and does not lead to cell damage, is currently not possible solely on the basis of electrophysiological criteria. In addition, the NUP may be experimentally of high amplitude, but may also be relatively shallow (Dreier et al., 2017). If the NUP is comparatively shallow, a cluster of SDs is typically superimposed on the NUP (Oliveira-Ferreira et al., 2010). This constellation indicates that only a fraction of the neurons in the tissue stays permanently depolarized, while the remaining fraction repolarizes after each SD. In contrast, the SD-initiated NUP in the wake of CA is typically of high amplitude and not associated with a cluster of superimposed SDs, as all neurons and astrocytes remain permanently depolarized and all cells will eventually die under this condition unless the tissue is reperfused in time.

## Conclusion

The animal experiments indicated that Pt/Ir electrodes on the brain surface can record 100% of SDs, SD-induced spreading depressions, non-spreading depression and SD-initiated NUPs, but negative DC reverberations following the recovery from SD may complicate evaluation, especially evaluation of the NUP. To identify a very slow DC potential component as NUP, the context with other factors such as initiation by SD, possible superposition by SDs, persistent depression of neuronal activity, decrease of rCBF and p_ti_O_2_, occurrence of a new focal or global neurological deficit, a new ischemic lesion in sequential neuroimaging scans, or death should also be considered.

In theory, epidural electrodes are a semi-invasive alternative to subdural recordings and can be viewed as an intermediate step between invasive subdural and non-invasive scalp EEG. However, in our patients, only 46% of subdurally recorded SDs and 28% of subdurally recorded SD-induced spreading depressions were also detected epidurally. Using epidural electrodes, the NUP may be easier to detect than SDs or SD-induced spreading depressions, but measuring the initial SD component and the SD-induced persistent depression helps to distinguish the NUP from artifacts, so subdural electrodes also offer metrological advantages in the case of a NUP. Within the limits of a small study, all this indicates that the protocol investigated here with two epidural electrodes cannot replace subdural electrode strips in the clinic. The next step in the development could be to place depth electrodes with several contacts epidurally to improve the yield. In addition, such arrays could also be tested subdurally, as this could still be less invasive than the placement of a subdural electrode strip. The goal must be to find the least invasive method that still provides robust and reliable data of sufficient quality to make clinical decisions. At present, it is the subdural electrode strip that meets these criteria and whose properties need to be exceeded.

## Ethics Statement

The animal study was carried out in accordance with the recommendations of the Landesamt für Arbeitsschutz, Gesundheitsschutz und technische Sicherheit Berlin (LAGetSi) (G0152/11). The protocol was approved by the Landesamt für Arbeitsschutz, Gesundheitsschutz und technische Sicherheit Berlin (LAGetSi) (G0152/11). All experimental procedures were conducted in accordance with the Charité Animal Welfare Guidelines. The reporting of animal experiments complies with the Animal Research: Reporting of *In Vivo* Experiments (ARRIVE) Guidelines. The clinical study was carried out in accordance with the recommendations of the ethics committee of the Charité – Universitätsmedizin Berlin (EA4/175/17) with written informed consent from the patients or the patients’ legally authorized representatives. All patients or patients’ legally authorized representatives gave written informed consent in accordance with the Declaration of Helsinki. The protocol was approved by the ethics committee of the Charité – Universitätsmedizin Berlin. Results are reported in accordance with the STrengthening the Reporting of OBservational studies in Epidemiology (STROBE) guidelines (https://www.strobe-statement.org).

## Author Contributions

JD and JW contributed substantially to the conception and design of the work, and drafted and revised the manuscript for important intellectual content. SM, CL, VK, CR, KS, NH, and JH drafted corresponding sections of the manuscript. All authors have approved the final version to be published and agreed to be accountable for all aspects of the work.

## Conflict of Interest Statement

The authors declare that the research was conducted in the absence of any commercial or financial relationships that could be construed as a potential conflict of interest.

## Footnotes

1 https://www.strobe-statement.org

## Abbreviations

AC: alternating current
ACSF: artificial cerebrospinal fluid
Ag/AgCl: silver/silver chloride
ARRIVE: Animal Research: Reporting of *In Vivo* Experiments
aSAH: aneurysmal subarachnoid hemorrhage
ATP: adenosine triphosphate
BBB: blood–brain barrier
BMBF: Bundesministerium für Bildung und Forschung (Federal Ministry of Education and Research)
CA: circulatory arrest
COSBID: Co-Operative Studies on Brain Injury Depolarizations
CT: computed tomography
CTA: computed tomography angiography
DC potential: direct current (steady) potential
DCD: organ donation after cardio-circulatory death
DCI: delayed cerebral ischemia
DFG: Deutsche Forschungsgemeinschaft
ECI: early cerebral ischemia
ECoG: electrocorticography
EEG: electroencephalography
EVD: extraventricular drainage
FiO_2_: fraction of inspired oxygen
ICP: intracranial pressure
ICU: intensive care unit
IQR: interquartile range
[K^+^]_o_: extracellular potassium concentration
KCl: potassium chloride
LAGeSo: Landesamt für Gesundheit und Soziales Berlin
LDF: laser-doppler flowmetry
MAP: mean arterial pressure
MCA: middle cerebral artery
MHS: malignant hemispheric stroke
MRI: magnetic resonance imaging
NUP: negative ultraslow potential
p_a_O_2_: arterial pressure of oxygen
p_a_CO_2_: arterial pressure of carbon dioxide
PTDDD: peak total SD-induced depression duration of a recording day
p_ti_O_2_: tissue partial pressure of oxygen
Pt/Ir: platinum/iridium
rCBF: regional cerebral blood flow
SD: spreading depolarization
SH: spreading hyperemia
SI: spreading ischemia
STROBE: STrengthening the Reporting of OBservational studies in Epidemiology
TBI: traumatic brain injury
TDDD: total depression duration per recording day
WFNS: World Federation of Neurosurgical Societies
